# Analysis of constraints and opportunities in maize production and marketing in Ethiopia

**DOI:** 10.1016/j.heliyon.2024.e39606

**Published:** 2024-10-18

**Authors:** Dagmawe Menelek Asfaw, Yibeltal Walle Asnakew, Fentahun Baylie Sendkie, Ahmed Abduletif Abdulkadr, Belayneh Asmare Mekonnen, Hailu Desalegn Tiruneh, Aden Mohammed Ebad

**Affiliations:** a Department of Economics, College of Business and Economics, University of Gondar, P.O. Box 196, Gondar, Ethiopia; b Department of Economics, College of Business and Economics, Injibara University, P.O. Box 40, Injibara, Ethiopia; c Department of Economics, College of Business and Economics, Samara University, P.O. Box 132, Samara, Ethiopia

**Keywords:** Constraints, Opportunities, Maize, Production, Marketing, Ethiopia

## Abstract

Maize, primarily composed of carbohydrates, is the cheapest source of calories. It plays a crucial role in domestic food supply, income generation, and employment. Additionally, maize constitutes a large share of the marketable surplus and is allocated a significant portion of cereal farmland. However, despite recent opportunities in production and marketing, the maize sector has faced various constraints, leading to significant declines in production, productivity, and marketable surplus in Ethiopia. By doing so, this study reviews the constraints and opportunities in maize production and marketing in Ethiopia, drawing from a range of sources including articles, documents, and workshop papers. Key constraints identified include limited access of improved seed varieties, irrigation, storage facilities, transportation, market place; outdated technology; poor pest management; market inefficiencies. In addition, soil degradation and land fertility issues combined with high input costs, delayed agricultural inputs, and insufficient credit and extension services hinder both production and marketing. Despite these challenges, there are notable opportunities for maize in Ethiopia. The crop thrives across diverse ecological zones, is resilient to various natural disasters, benefits from fertile soils, regular rainfall, a stable environment, and abundant water resources. Additionally, favorable government policies, increasing demand for maize, and the expansion of agro-processing industries present significant opportunities. To enhance maize production and marketing in Ethiopia, several actions are recommended, for instance, improving land tenure security, developing rural infrastructure, conserving natural resources and the environment, providing modern agricultural inputs, stabilizing maize prices, reducing transaction costs, and promoting agricultural extension services.

## 1 Introduction

Maize (*Zea mays*), commonly known as corn, is one of the world's most widely cultivated cereal crops. Originating from Central America, maize has become a crucial food staple globally due to its versatility and high nutritional value. It serves as a primary food source for billions of people and is used in various forms, from whole grains to processed foods and animal feed. The crop’s significance extends beyond human consumption; it plays a critical role in global agriculture, contributing to the livelihoods of millions of farmers and supporting economies worldwide [1].

The global maize industry faces several challenges, including climate change, which affects weather patterns and increases the frequency of extreme weather events. Additionally, pests and diseases pose significant threats to maize production, impacting yields and quality. However, there are also opportunities arising from technological advancements, such as genetically modified varieties and improved agricultural practices, which aim to enhance productivity and resilience [2].

In Sub-Saharan Africa, maize is a staple food and a key component of food security. The region is heavily reliant on maize for dietary energy, making it integral to the daily lives of millions. Despite its importance, maize production in Sub-Saharan Africa faces numerous constraints. Smallholder farmers, who dominate maize cultivation, often struggle with low productivity due to factors such as inadequate access to modern agricultural inputs, limited technical knowledge, and challenging environmental conditions [3].

Climate variability, including droughts and irregular rainfall, further exacerbates the difficulties faced by farmers. Additionally, infrastructure deficits, such as poor road networks and insufficient storage facilities, hinder market access and reduce the profitability of maize farming. Despite these challenges, there are emerging opportunities for improving maize production and marketing in the region, driven by initiatives aimed at enhancing agricultural practices, increasing access to technology, and fostering regional trade [4,5].

Ethiopia is one of the largest maize producers in Africa, with the crop being a major staple food and a critical component of the national diet. Maize cultivation is widespread across the country, encompassing various agro-ecological zones that offer diverse growing conditions. The crop plays a central role in Ethiopia's agriculture, which employs a significant portion of the population and contributes substantially to the national economy [4].

Maize plays a crucial role in Ethiopian agriculture, with approximately 8.96 million smallholder farmers engaged in its cultivation. This crop covers about 2.526 million hectares of cereal farmland in the country, with average yield of 4 metric tons per hectare in 2022. Notwithstanding, this productivity relatively low compared to global standards, where average maize yields can exceed 5 metric tons per hectare [6]. In 2019, maize production for Ethiopia was 8.5 million metric tons with growing at an average annual rate of 7.64 % [7]. However, in 2022, maize production faced challenges again with estimates dropping back down to about 7.8 million metric tons due to adverse weather conditions including droughts in some regions and ongoing issues related to supply chain disruptions caused by global events [8].

Of the total grain produced in Ethiopia, approximately 72 % is consumed domestically, while the remaining 31 % of the grain production, which includes oilseeds and pulses is marketed. Notably, maize represents 19.2 % of this marketed portion, resulting in a marketable surplus of 20.1 % for maize in 2022 [7].

In terms of calorie intake maize is the cheapest source of calorie, at a price of 25 ETB per kilogram (1000 g), maize costs about 0.068 ETB per calorie (25 ETB /(3650 calories). In contrast, rice, which provides about 365 kilocalories per 100 g, costs roughly 0.11 ETB per calorie, and wheat flour, providing about 340 kilocalories per 100 g, costs about 0.14 ETB per calorie. In addition in term of domestic food supply, income generation and source of employment maize is the most important staple crop for the rural Ethiopian population [9]. Maize is primarily composed of carbohydrates, making it a significant source of energy. On average, maize contains about 70–75 % carbohydrates; Maize has a relatively low fat content, around 4–5%. The fats are mostly unsaturated, which is beneficial for health. In Ethiopia, maize is often consumed as porridge (known as "kita" or "genfo"), flatbread (similar to injera), or as a component of various traditional dishes. It is a primary source of calories for many Ethiopians [10,11].

In such a way, the opportunities for maize production in Ethiopia are substantial. The country has favorable agro-ecological conditions, government support, and technological advancements that can drive growth in maize production. Expanding cultivation, improving productivity through modern practices and research, tapping into domestic and regional markets, and enhancing climate resilience are key areas that offer significant potential for maize production in Ethiopia. Continued investment in these areas will be crucial for realizing these opportunities and achieving sustainable growth in maize production [12].

Even though, the existence of such decent opportunity and advancements in agricultural technology, maize production, productivity and market share tends to reduce over time. For instance, the productivity in 2022 was 4 metric tons per hectare, which was relatively low compared to global standards [6]; maize production for Ethiopia was decreasing from 8.5 million metric tons in 2019 to 7.8 million metric tons in 2022 [8]; maize’s share in cereal production is roughly decrease from 23 to 19 % in 2022 [7]. Despite of this, existing literatures like [5,6,[11], [12], [13], [14], [15], [16], [17]] and others has analysis several aspects of maize production as well as marketing separately, including agronomic practices, market access, and socio-economic factors affecting farmers. However, there remains a lack of comprehensive studies that integrate opportunity and challenges of maize production and marketing. Therefore, this study provides a comprehensive analysis of the challenges and opportunities associated with both maize production and marketing in Ethiopia by reviewing different empirical evidence.

## 2 Objectives of the review

This review basically looks over constraints and opportunities of maize Production and marketing in Ethiopia by addressing the specific issues on.

➢ To review the maize productivity and production trends in Ethiopia;
➢ To review maize production and marketing constrained in Ethiopia; and
➢ To review the opportunities for the production and marketing of maize in Ethiopia

## 3 Research questions

➢ What are the historical and current trends in maize productivity and production in Ethiopia?
➢ What are the key constraints affecting maize production in Ethiopia?
➢ What are the main challenges faced in the marketing of maize in Ethiopia?
➢ What opportunities exist for enhancing maize production in Ethiopia?
➢ What are the potential market opportunities for maize in Ethiopia?

## 4 Methodology

### 4.1 Study design and searching strategy

To make review on constraints and opportunities of maize Production and Marketing in Ethiopia, we conducted a systematic literature study design from different academic search engine: Web of knowledge (apps.webofknowledge.com), Scopus and Science Direct, Research Gate (https://www.researchgate.net), Google Scholar (scholar.google.com), PubMed and other grey literature by several keywords were chosen to obtain a wide number of search results. All keywords were searched in Ethiopia as well. The keywords selected were: “Opportunities and maize Production”, “Opportunities and maize Marketing”, “Constraints and maize Production”, “Constraints and maize Marketing”, Constraints and Opportunities of maize Production, Constraints and Opportunities of maize Marketing. The literature included mainly peer-reviewed articles, but sometimes also a few studies in technical journals, books, unpublished papers and conference proceedings. From the total 115 papers founded based on such search strategies, however only 38 paper has been reviewed based on eligibility criteria’s between January 2024 and June 2024.

### 4.2 Eligibility criteria

#### 4.2.1 Inclusion criteria

• All observational studies, including cross-sectional, time series, panel, cohort and descriptive studies.
• All articles published only in the English language and published and reported between 2014 and 2024.
• All articles which were done in Ethiopia and reporting the challenges and opportunity of production and marketing of maize production in Ethiopia.

#### 4.2.2 Exclusion criteria

• Articles without full text and data that are difficult to extract
• Studies published in languages other than English and published and reported before 2014.
• All articles which were done out of Ethiopia and does not reporting the challenges and opportunity of production and marketing of maize production in Ethiopia.

## 5 Literature reviews

Under this part we have been reviewing the production trend and productivities, production and marketing constrained, production and marketing opportunities of maize production in Ethiopia based on different peer reviewed articles, books, unpublished papers and conference proceedings.

### 5.1 Maize productivity and production trends in Ethiopia

Agriculture in Ethiopia is the mainstay of its economy and employs opportunity for Ethiopian population. The mainstreams of employees in agricultural sector are smallholder farmers by own less than 1 ha of land practicing subsistence farming on it. Those farmers were primarily produced cereal crops, which are account for 95 % of the total agricultural production in Ethiopia. Cereals are the major crops produced in the country and they constitute the largest share of domestic food production [18]. From those crops five major cereals (teff, wheat, maize, sorghum, and barley) are the main engine for Ethiopia’s agriculture and food economy, accounting for about three-fourths of the total area cultivated, 39 percent of agricultural gross domestic product (GDP) in 2020/21, 13.5 percent of total GDP, and 61 percent of calories consumed [8,19]. There has been substantial growth in cereals in terms of area cultivated, yields, and production since 2019, but yields are low by international standards [4].

According to Ethiopian Statistics Service (ESS) [7] report that in 2022 main cropping season, cereals were cultivated on 9.9 million hectares of land, this represented 69.1 % of total area allotted for agricultural production. Likewise, during the same year, 21.5 million tons of cereal were produced, which is also 71.2 % of total food grains production of the country. Based on land coverage, the ranking of cereals in Ethiopia is as follows: teff occupies 30.8 % of the total land allocated for crops, maize covers 22 %, wheat accounts for 19.9 %, and sorghum occupies 14.4 %. In terms of total production, maize leads with 32.5 % of the total crop production, followed by wheat at 22.3 %, and teff at 19.8 %. Regarding productivity, maize ranks second only to rice, with a production of 4 metric tons per hectare, outperforming other crops (see Table 1).

**Table 1:** Main cereal production area, total production and productivity in Ethiopia for the main-season in 2022.

Crops	Area ('000 Hectare)	Production ('000 Tone)	Yield (Tone/Hectare)<
Barley	970	2400	2.47
Maize	2550	10200	4.00
*Teff*	3562^Ϯ^	6212 ^Ϯ^	1.74 ^Ϯ^
Sorghum	1660	4200	2.53
Wheat	2300	7000	3.04
Millet	455	1150	2.52
Oats	24.6	53.7	2.18
Rice, paddy	34	208	5.99

Maize stands out as the most widely produced and consumed cereal in Ethiopia, with the largest production volume and area cultivated compared to other cereals. While teff is culturally and nutritionally significant, it occupies a smaller share of total production. Wheat and sorghum also play important roles but with different production scales and consumption patterns. Each cereal crop has its unique role in Ethiopian agriculture, food security, and economy, with ongoing efforts to address challenges and leverage opportunities for growth.

Increasing maize yield and reducing the yield gap are essential to ensure future food security in Ethiopia [21]. Overall, maize productivity and production in Ethiopia have shown substantial improvements, largely attributed to the expansion of cultivation areas, the introduction of new maize varieties suited to diverse environmental conditions, and the increased awareness fostered by government extension services. Additionally, maize plays a crucial role in food security due to its low cost as a caloric source compared to other major cereals and its affordable price per kilogram [14]. In doing so, maize production in Ethiopia has increased in the past decade because of both an increase in production area and productivity [21].

**Fig. 1:**
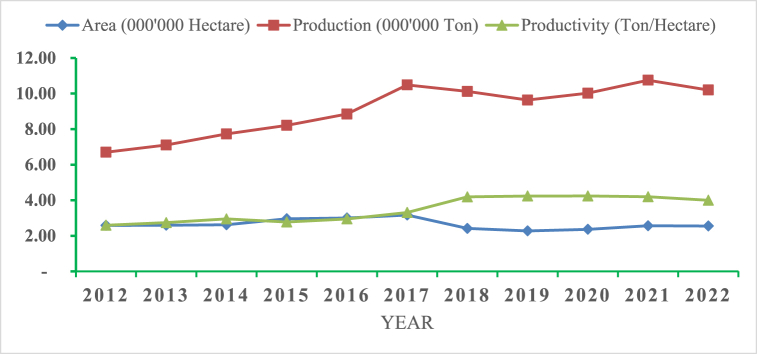
Production area, total production and productivity of maize in Ethiopia from 2012 to 2022.

The area dedicated to maize cultivation shows a generally increasing trend from 2012 to 2016, peaking at 3.00 million hectares in 2016. After that, the area under maize cultivation fluctuated slightly, with a noticeable decrease in 2018 and 2019, and a partial recovery in 2020 and 2021, but a slight decline again in 2022.The variability in the area under cultivation might reflect changes in land allocation priorities, environmental conditions, or shifts in agricultural policies (see Fig. 1).

Source [20]:

Maize production increased from 6.70 million tons in 2012 to a peak of 10.75 million tons in 2021. There was a slight decline to 10.20 million tons in 2022.The overall upward trend in production suggests improved farming practices, better seed varieties, or increased investment in maize cultivation. The slight dip in 2022 could be due to various factors, such as adverse weather conditions or market dynamics (see Fig. 1).

Productivity, measured in tons per hectare, showed a significant increase from 2.59 tons/hectare in 2012 to a peak of 4.24 tons/hectare in 2018 and 2019. However, productivity slightly declined to 4.00 tons/hectare in 2022.The rise in productivity indicates that technological improvements, better agronomic practices, and enhanced input usage have contributed to higher yields per hectare. The slight decline in recent years might warrant further investigation into possible issues like soil fertility, pest problems, or resource management In addition, it may be due to the reason that, technical efficiency yield gap(gaps in knowledge, information and skill), the economic yield gap (farmers from using the often large amount of inputs to achieve potential yield) [21], low profitability [9], post-harvest losses [11,22] (see Fig. 1).

### 5.2 Maize production and marketing constrained in Ethiopia

In Ethiopia, the agricultural sector, particularly cereal production and marketing, is the largest sub-sector in several key aspects. It provides job opportunities for 60 % of rural dwellers, occupies 88 % of the total grain area, and contributes to 88 % of total grain crop production. Additionally, it accounts for more than 40 % of a typical household’s food expenditure, 60 % of total caloric intake, and 30 % of the national GDP [15,23]. However, of the total cereal production, excluding the portion set aside for consumption, only 28 % of the total grain production (including oilseeds and pulses) is marketed [24].

Maize is one of the most important staple crops of the country in terms of production, yield, affordable unit price and cheapest caloric source (20 percent of total caloric) [25]. Even though, marketing and production of cereal in general maize in particular were devastated, for instance according to the report of FAO [20] the yield or productivity of maize 4 metric ton per hectare, which was less than water-limited yield potential of maize in Ethiopia (12.5 metric ton per hectare) and also this productivity relatively low compared to global standards, where average maize yields can exceed 5 metric tons per hectare [6]. A study by Alemu, Yirga [9], found that about 18 percent of maize producers participate in maize markets, which is lower than Teff (48 percent) and wheat producers (37 percent). In such a way, it is compulsory to investigate and review the major production and marketing constrained of maize production in Ethiopia.

Maize producers face several challenges, including a limited availability of enhanced maize varieties, outdated technology, season-dependent conditions, and weather-related problems. Additional issues include insufficient irrigation, pest and disease problems, inadequate credit services, and a shortage of market information. Furthermore, there are difficulties related to inadequate storage facilities, ineffective post-harvest management, unregulated dealers, insufficient market knowledge, price fluctuations, and poor maize quality were long-standing constraints production and marketing of maize [13,26].

In order to transport maize inputs and output to and from farms road and market development infrastructure were a vital, even though road and market development infrastructure were inadequate and poor conditions in Ethiopia [23]. The report of FAO [1] emphasize that that due to lack of basic infrastructures of bridge between rural and urban areas, the growth of agro-industry unable to achieve it potential level of development. World Bank [24] and Alemu, Berihun [22] found that, there was a shortage of storage for Producers, cooperatives, traders and wholesalers, which makes the producers losses of output during storage due to weevil, rodents and fungus discourage farmers. And also cooperative unions are not able to buy all their maize output from the farmers due to limited storage and working capital, in addition financial constraints and poor market linkages also another constrained.

According to the Abebe and Halala [14,16], there is a significant shortage of storage facilities for producers, cooperatives, traders, and wholesalers. This lack of adequate storage leads to losses of maize due to damage from weevils, rodents, and fungus, which discourages farmers. Additionally, financial constraints and weak market linkages further exacerbate the issue.

Willy [27] investigated constraints and opportunities of maize, teff and wheat production at Ambo and Toke Kuttaye districts on 180 sample farmers found that the basic constraint are crop worm and disease, price problem means that high for inputs and low for products, limited availability of inputs, and lack of appropriate threshing facilities and storage facilities which causes high post-harvest losses. Yirga, Nin-Pratt [28] and Newman [5] proposed that the factors limiting maize productivity in Ethiopia include insufficient access to improve or hybrid seeds, inadequate seed multiplication capacity, and low profitability and efficiency in fertilizer use. These issues are compounded by the absence of complementary improved practices and seeds, as well as challenges related to irrigation and water availability.

A study by Tuki [15], found that participating in off-farm activities, being female household headed and having access to credit were decrease the tendency of farmer to supply maize to the market, whereas, availability of market nearest to farmer residents, gets formal education or training, having access of larger size of land, being more experience were the main factors that was increasing marketable surplus of maize. This finding also supported by Abebe and Halala [16], which was found that as market land size of the household increase by 1 ha, volume of market supply increased by 1.3 %.

Heckman two stage model by Abebe and Halala [16] found that, unstable price, weak market information, presence of non-licensed traders and absence of grades and standards, limited access to credit, less amount of improved seeds usage per hectare, low pesticide application, low levels of irrigation, soil degradation and soil erosion, inadequate agricultural research and extension and poor coordination among traders were the major market constraints that hinder maize production and marketing in Wolaita and Dawuro zones, Ethiopia.

The fertility and stability of farmland play a crucial role in enhancing productivity, supporting plant growth, and optimizing crop yields, including maize. However, in Ethiopia, land fertility has been compromised. In the highlands, soil erosion is a significant issue, while in the lowlands, land degradation is exacerbated by deforestation, overgrazing, excessive tree cutting, shifting cultivation, and poor management of soil and water resources [14]. Problems such as inadequate adoption of soil and water conservation practices, improper crop rotation, use of marginal lands, unbalanced fertilizer application, mismanagement of irrigation systems, and over-extraction of groundwater have all contributed to reduced land fertility. These factors have been identified as constraints to maize production and yield in Ethiopia [29,30].

During the harvest season, farmers often sell their maize quickly at reduced prices to cooperatives, wholesalers, and retailers to settle debts and cover other expenses. As a result, they do not see significant profits from their maize harvest and become less motivated to harvest it. This issue arises because farmers lack access to adequate storage facilities to hold their maize until prices are higher and also struggle with limited access to credit to manage their financial obligations [30,31].

When the farmers commercialized their product like maize, there incurred transaction costs (fixed transaction costs^1^ and proportional transaction costs^2^). At this time the farmer were obligated to choice either receiving below market prices or incurring high costs when searching for better prices. Those higher transaction cost exists because of smallholder maize farmers have poor and asymmetric access to information, and markets are poorly coordinated [32]. However the increments of such transaction costs are the critical challenges to smallholders maize farmer for participating in marketing [33,34].

Delayed deliveries of agricultural inputs, absence of credit markets, and lack of access to agricultural technology are vital maize production constraint in Ethiopia [30,35]. For example according to the report of FAO [36], an average distance of 44 km to the closest road isolates many farmers from more lucrative markets. Due to these, the majority of farmers sell their production in local markets or other informal channels, this leads to income losses and less enthusiastic to participate in the market.

The study conducted by reveal Rashid, Getnet [12] that, the major constraint of maize production were price fluctuation of maize grain in the maize market, which was agreed by more that 80 percent of total respondents. Another constraint factor in maize production and marketing in this study area were low soil fertility, high input prices especially fertilizers and seed, diseases on the biotic and abiotic, weeds, labor shortage (especially in harvest season) and early cessation of rain. This result supported by Refs. [11,21].

In a study on crop production and marketing challenges in Bench-Sheko by Tadesse, Tilahun [13] utilized a stochastic frontier model to identify major issues affecting maize production. These included farmers' reluctance to adopt improved technologies, insufficient availability and use of improved seed varieties (94.5 %), inadequate fertilizer supply and use (95 %), gaps in farmers' knowledge and skills (80.1 %), poor extension services (57.3 %), soil acidity (94.8 %), pests and diseases (77.8 %), conflicts (84.9 %), and outbreaks of human diseases (60 %).

Additionally, Newman [5] findings indicate that both maize production and marketing face risks and costs that threaten food security. Production costs are linked to technological applications, while weather risks affect availability. Market-related issues such as inadequate rural infrastructure including storage, roads, telecommunications, and institutional support contribute to higher consumer food costs and restrict the benefits farmers can gain from commercialization.

### 5.3 Opportunities for the production and marketing of maize in Ethiopia

Maize production in Ethiopia is poised to benefit from several key opportunities. The availability of advanced seed varieties and hybrid crops, coupled with improved agricultural practices, can enhance yields and resilience. Increased investment in infrastructure, such as irrigation and storage, alongside strengthened agricultural extension services, will support better farming techniques and reduce post-harvest losses. Supportive government policies and growing domestic and regional demand for maize further provide favorable market conditions. Additionally, ongoing research and development in agricultural technologies, along with climate adaptation strategies, promise to boost productivity and sustainability in maize farming [5].

Despite challenges, favorable land and climatic conditions, along with significant productivity potential, present opportunities for maize production in these areas. Maize serves as a primary cash crop and food source for farmers in various lowland and mid-altitude regions of Ethiopia, supporting both subsistence and commercial farming. This potential has led to increased maize production in these zones [16]. Similarly, the existence of strong multipurpose farmer’s cooperatives, using maize for multipurpose in the area and its productivity per hectare were the major opportunities in Dembecha District, Ethiopia [14].

Research initiatives such as the National Maize Research Project and the development of hybrid seeds from companies like Corteva-Pioneer, coupled with increased investment in extension services, demonstrate that maize production has the potential to serve as a model for scaling up agricultural productivity through innovative practices, ultimately contributing to food security (USDA, 2020). In contrast, Van Dijk, Morley [21] highlighted that the expanded availability and use of modern inputs, such as advanced seed varieties and fertilizers, along with improved extension services and rising demand, present significant opportunities for enhancing maize production and marketing in Ethiopia.

Ethiopia has fertile soil, regular rainfall suitable environment and water resources availability and suitability for irrigation to produce different agricultural outputs like maize. Therefore such fertile soil, regular rainfall, favorable environment and availability of irrigated land are better situations as opportunities for maize production in Ethiopia [27,37].

Maize in Ethiopia grow across different ecological zone, highly tolerable for environmental change like drought, high yield and productivity [14], resistance to disease and insect pests, lodging resistance, ability to perform well under low soil fertility, and a combination of these factors were considered as good opportunity for maize production. Likewise, increase the availability of fertilizer, improved maize seed, development of widely adapted and profitable varieties and hybrids, awareness created by government extension support, availability of moisture during the growing season, soil conservation are enthusiastic for both production and marketing of maize in Ethiopia.

The aggregate demand for maize has seen significant shifts over time, largely due to several factors. Increasing populations in both urban and rural areas have driven demand, as maize is generally less expensive compared to other cereals like Teff [14,16]. Additionally, maize serves as a vital and cost-effective source of calories (CSA, 2019; Rashid, 2010). Supportive government policies that promote private sector involvement, develop market linkages, and offer incentives such as investment land and tax exemptions have also played a crucial role in driving the growth of the maize market [1].

Maize is an importance ingredient for agro processing industries like mill and flour factories, production of starch for textile industries, production of edible oil for domestic purposes [10], in a line with this currently in Ethiopia agro processing industries are expanding radically, increasing the participation of private sector from production to distribution and strong multipurpose farmer cooperatives are also responsible for the expansion of maize market and production.

## 6 Limitations of the study and areas for further research

The review of constraints and opportunities in maize production and marketing in Ethiopia reveals several limitations that warrant consideration. Firstly, the study may be constrained by a lack of comprehensive data across different countries, which can lead to an incomplete understanding of the diverse challenges faced by farmers. Additionally, the reliance on secondary data sources might introduce biases or inaccuracies, as these sources may not reflect the most current conditions or practices in maize production. Furthermore, socio-economic factors such as gender roles and access to resources are often underrepresented in agricultural studies, limiting insights into how these dynamics affect maize production and marketing.

For future research, it is essential to conduct primary data collection through surveys and interviews with local farmers to gain a more nuanced understanding of their experiences and challenges. Investigating the impact of climate change on maize yields and exploring innovative agricultural practices could provide valuable insights into enhancing productivity. Moreover, examining market access issues, including infrastructure development and policy implications, would contribute significantly to understanding how to improve maize marketing strategies in Ethiopia. Lastly, integrating gender analysis into agricultural research could uncover critical barriers faced by women farmers and help design targeted interventions.

## 7 Conclusion and recommendations

### 7.1 Conclusion

Maize is a crucial staple crop in Ethiopia, contributing 32.5 % to the total crop production, with an average yield of 4 metric tons per hectare. It is valued for its affordability and serves as a key source of calories, accounting for 20 % of total caloric intake. Maize plays a significant role in ensuring food security, providing employment, supplying raw materials for agro-processing, and covering approximately 88 % of household food consumption. Over the past two decades, the maize sector has shown remarkable growth in production, yield, and marketing.

However, recent years have seen a decline in maize production and marketing performance due to various constraints issues including limited access to improved seed varieties, outdated technology, inadequate irrigation, and poor pest management. Farmers struggle with inadequate storage facilities leading to high post-harvest losses and financial constraints, while poor infrastructure hampers transportation and market access. Market inefficiencies, such as price fluctuations, weak market information, and the presence of unlicensed traders, further exacerbate the problem. Soil degradation and land fertility issues also contribute to reduced productivity. Overall, these factors combined with high input costs, delayed agricultural inputs, and insufficient credit and extension services, hinder both production and marketing.

Despite these challenges, there are promising opportunities for the maize sector. Government policies are fostering investment in market infrastructure, reducing controls, and expanding social safety nets. Additionally, there are increased investments in extension services, development of improved and hybrid maize seeds, and better access to modern inputs such as fertilizers and pesticides. Favorable conditions, including fertile soil, regular rainfall, and suitable growing environments across various ecological zones, further enhance the sector's potential. Rising market demand driven by population growth, agro-processing industry development, and competitive pricing also offer significant prospects for maize production and marketing in Ethiopia.

### 7.2 Policy recommendations

Based on above intensively reviewing of different source on maize production and marketing, forwarded the following policy recommendation for responsible bodies.

➢ The government should develop and implement land legal tender framework for proper land properties and tender management.
➢ Investment should be done on infrastructural development like rural to urban road, storage facilities, developed nearby market infrastructure.
➢ Modern agricultural inputs (fertilizer, insecticide, herbicide, improved maize seed) should be provided in relatively lower cost and abundant quantity on time and make awareness to develop the habit of using modern inputs and irrigation practice to the society by agricultural extension.
➢ Any responsible bodies including government should participate the work of natural resource and environmental conservation.
➢ The government should help the maize producer by stabilizing the unit price of maize specially during harvesting season, reducing transaction cost (like providing market information) and sustained profitability.

## CRediT authorship contribution statement

**Dagmawe Menelek Asfaw:** Writing – review & editing, Writing – original draft, Resources, Methodology, Investigation, Funding acquisition, Formal analysis, Data curation, Conceptualization. **Yibeltal Walle Asnakew:** Writing – review & editing, Writing – original draft, Supervision, Methodology, Investigation, Data curation, Conceptualization. **Fentahun Baylie Sendkie:** Writing – review & editing, Validation, Supervision, Investigation, Data curation. **Ahmed Abduletif Abdulkadr:** Writing – original draft, Visualization, Supervision, Methodology, Investigation, Formal analysis. **Belayneh Asmare Mekonnen:** Visualization, Supervision, Funding acquisition, Data curation, Conceptualization. **Hailu Desalegn Tiruneh:** Writing – review & editing, Project administration, Formal analysis, Data curation, Conceptualization. **Aden Mohammed Ebad:** Writing – review & editing, Visualization, Investigation, Formal analysis, Data curation, Conceptualization.

## Availability of data and materials

The datasets and articles used to support this study are available from the corresponding author upon reasonable request.

## Consent for publication

All the authors given consent to publish.

## Ethics approval and consent to participate

This review does not require permission from ethical committee.

## Funding

There was no funding support during this study.

## Declaration of competing interest

The authors declare that they have no known competing financial interests or personal relationships that could have appeared to influence the work reported in this paper.

## Footnotes

1 Costs associated with information, negotiation and monitoring.

2 Costs of transferring the products or inputs being traded such as transportation costs.
